# Transgenerational Epigenetic DNA Methylation Editing and Human Disease

**DOI:** 10.3390/biom13121684

**Published:** 2023-11-22

**Authors:** Joshua D. Tompkins

**Affiliations:** Department of Diabetes Complications and Metabolism, Arthur Riggs Diabetes and Metabolism Research Institute, City of Hope, Duarte, CA 91010, USA; jtompkins@coh.org

**Keywords:** DNA methylation, epigenetics, epimutation, heritable, transgenerational, development, epigenetic editing, cytosine, dCas, germline

## Abstract

During gestation, maternal (F0), embryonic (F1), and migrating primordial germ cell (F2) genomes can be simultaneously exposed to environmental influences. Accumulating evidence suggests that operating epi- or above the genetic DNA sequence, covalent DNA methylation (DNAme) can be recorded onto DNA in response to environmental insults, some sites which escape normal germline erasure. These appear to intrinsically regulate future disease propensity, even transgenerationally. Thus, an organism’s genome can undergo epigenetic adjustment based on environmental influences experienced by prior generations. During the earliest stages of mammalian development, the three-dimensional presentation of the genome is dramatically changed, and DNAme is removed genome wide. Why, then, do some pathological DNAme patterns appear to be heritable? Are these correctable? In the following sections, I review concepts of transgenerational epigenetics and recent work towards programming transgenerational DNAme. A framework for editing heritable DNAme and challenges are discussed, and ethics in human research is introduced.

## 1. The Epigenome and DNA Methylation

**Figure 1:**
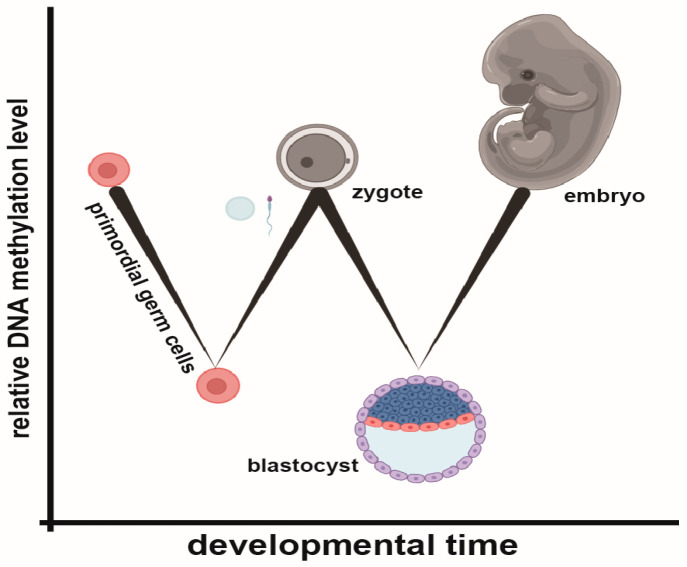
An overview of DNAme through developmental time. Massive demethylation waves occur in primordial germ cells and pre-implantation stages of development, followed by stage-specific DNAme reacquisition.

The epigenome functions to regulate DNA interactions and provides a basis for the storage and extraction of information across the genome. Radiating outward from a single cell, each of the trillions of derivative cells will adopt a unique epigenome, similar by cell and tissue type, responsive, and reinforced by an array of epigenetic regulators. Major epigenetic factors include covalent DNA modifications, such as DNA methylation (DNAme) or quadruplex structures, a multitude of histone modifications and chromatin remodelers, which facilitate DNA accessibility, as well as short- and long-noncoding RNAs that mediate DNA-protein interactions [1,2,3,4,5]. There are some cells which maintain epigenome plasticity over their lifetime (e.g., multipotent stem cells), while others exhibit terminal post-mitotic configurations (e.g., cardiomyocytes) [6,7,8,9,10,11,12]. Primordial germ cells (PGCs) and cells of the pre-implantation blastocyst stage of development will uniquely undergo a remarkable form of epigenetic remodeling, in which genome presentation is reset, and the genome evolves between parent and child [11,12,13,14,15]. During these stages, DNAme is globally removed. Yet, the decoration is again re-established during gametogenesis and blastocyst stages of development (Figure 1).

**Figure 2:**
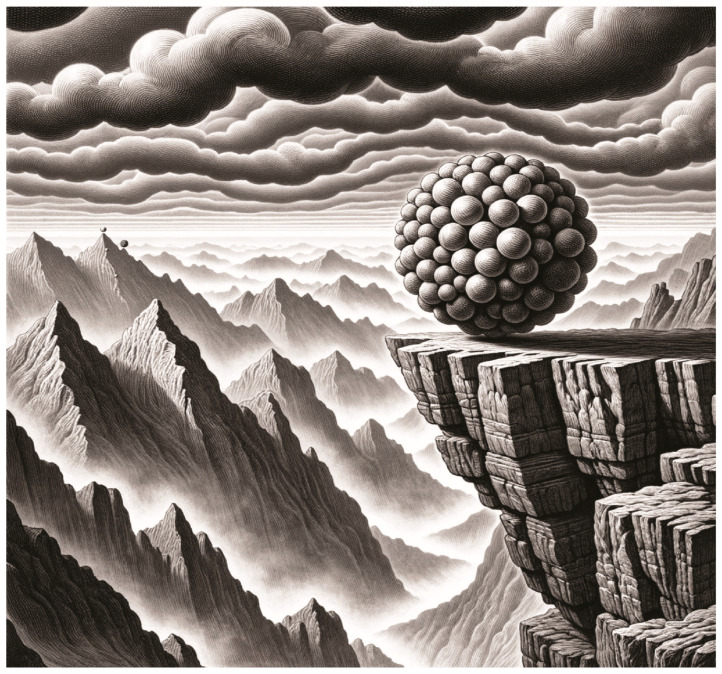
An epigenetic landscape of developmental potential generated with the help of AI DALL-E3 (OpenAI, San Francisco, CA, USA). The ball of pluripotent stem cells represents early developmental potential, where fate is intrinsically regulated by the epigenetic landscape that awaits as it rolls forward in developmental time. DNAme can be visualized as a looming fog, which accumulates over developmental time and clouds cellular potential, as cells transverse narrowing valleys. Auxiliary forces visualized by a growing storm of clouds may erode and re-contour this potential. Migrating PGCs are illustrated moving along a rare path to a distal peak and escaping the downward forces of somatic cell development, transversing upward onto the next generation’s highest developmental potential. Next-generation ESCs are displayed as well as another developing blastocyst. This feat becomes more difficult when cells have accumulated toxic transgenerational DNAme information.

It was Conrad Hal Waddington who first defined epigenetics as the “branch of biology that studies the causal interactions between genes and their products which bring the phenotype into being” (p. 5, [16]). Scientists would later ascribe an array of epigenetic modifications and editors that progressively restrict developmental potential in much the same way Waddington viewed epigenetic forces must exist, most famously illustrated by his epigenetic landscape. Indeed, the cellular potential is restricted over developmental time, much like a ball driven by gravity transversing a series of narrowing valleys finds an ultimate pathway (Figure 2). An image has been generated with the help of DALLE-3 artificial intelligence (AI) to conceptually illustrate this, with certain updates for current developmental biology. A ball of embryonic stem cells, perched at the edge of a cliff overseeing valleys of fate, having rolled slightly downward from the totipotent zygote, would fall stochastically forward in developmental time, dividing and spilling ultimately trillions of cells into the valleys below. The fog represents DNAme accumulating over developmental time and the corresponding removal of developmental potency. A somatic cell will ultimately become clouded in its identity. If we imagine a rare group of cells, uniquely protected from commitment and finding a rare pathway across the highest ridgelines, this would be akin to primordial germ cell migration and the maintenance of epigenomic potential between generations, as discussed in the following sections. Extrinsic forces may recontour these ridges and valleys, some of which are required in normal balance (e.g., maternal hormones), and some of which may be pathological (e.g., gestational diabetes).

Though Waddington did not refer to contemporary features we now understand to be epigenetic, key epigenetic factors, including methylated DNA, were, in fact, discovered prior to his work. Cytosine methylated DNA was originally synthesized by Treat Johnson in 1904, and he would use the corresponding crystal picrate to later identify 5-methylcytosine (5mC) in bacteria. He would write, “the discovery of this compound increases the number of pyrimidines functioning in life” [17,18]. However, eukaryotic detection and epigenetic implications would take several additional decades of work. Observations of asymmetric DNAme across cell types and species, and the identification of DNA methylating and demethylating systems, represent pivotal moments in epigenetic discovery. “The amounts [of 5mC] in which it occurs, however, varying with the source but constant from a given source, suggest that it is an essential constituent of certain DNAs and no accident of enzyme action”, Wyatt 1951, (p. 583, [19]). DNAme has since become the most extensively studied epigenetic mark, owing to its early discovery, demonstrated requirements for mammalian development, and long-term stability in archived DNA samples [20]. References for additional historical insights on epigenetic concepts and DNAme discovery are provided [2,20,21].

## 2. DNA Methylation in Development, Cell Memory, and Disease

**Figure 3:**
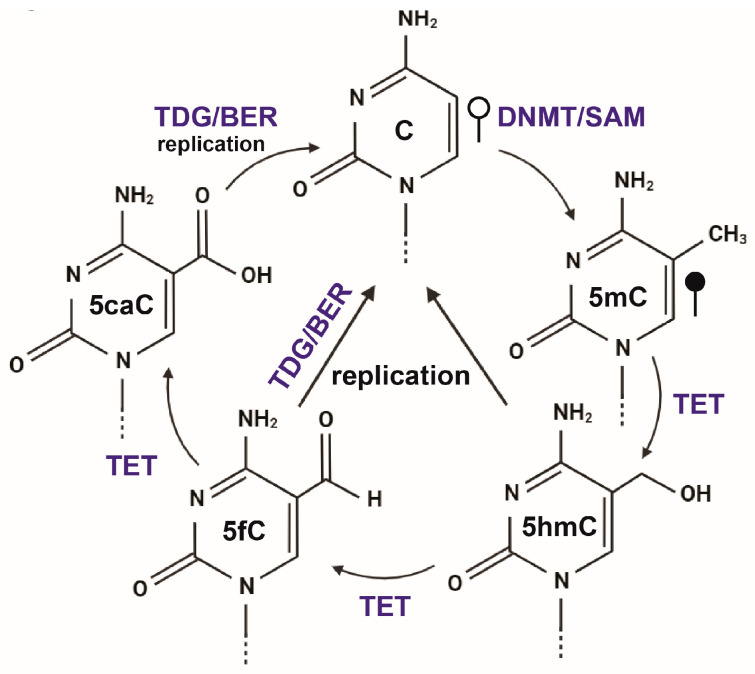
An overview of cytosine DNAme and demethylation is provided. Briefly, C becomes methylated at C5 with DNMT activity and co-factor S-adenyl-methionine (SAM). 5mC can be actively oxidized to 5-hydroxymethylcytosine (5hmc), then to 5-formylcytosine (5fC), and 5-carboxylcytosine (5caC) intermediates, which can be removed either actively by Thymine DNA glycosylase (TDG) and base excision repair (BER), or passively through DNA replication.

Epigenome configurations change in response to developmental cues, often drastically, and coordinate the differentiation and function of cells as they integrate multiple complex organ systems. DNAme is required for cellular differentiation [11,12,22,23]. The enzymatic reaction occurs at the C-5 position of cytosine, predominately in CpG contexts, which are often functionally clustered together in CpG-dense regions termed CpG “islands” (CGIs) [20]. These are speckled across an evolutionarily CG-depleted genome. Three enzymes are known to form 5mC. DNMT3A and 3B’s de novo methyltransferase activity is reinforced by maintenance enzyme DNMT1, which positively associates with hemimethylated DNA and faithfully copies DNAme patterns onto the nascent strands during DNA replication [24]. On the other hand, three enzymes, TET1, 2, and 3, actively demethylate DNA via 5mC oxidation to 5-hydroxymethylC and potential removal via subsequent base excision repair or passively through DNA replication (Figure 3) [25]. For the most part, genome-wide DNAme patterns are stably maintained; however, during cellular differentiation, DNAme patterns are dynamically altered [9,10,26]. Here, DNAme represents a conserved system for transcriptional regulation and cell memory, which protects the genome from transposable elements and enables the specification of unique cellular identities [6,9,11,12,27,28,29,30,31]. For example, developmental transcription factors become gene body hypermethylated upon activation and retain residual DNAme signatures, and DNAme directs Polycomb repressive activity in chromatin organization during the earliest stages of development [9,13,32]. Such activities are also found at developmentally poised promoters and enhancer networks, which may be particularly dynamic prior to the specification of cellular fate [33,34,35].

There are times when pathological DNAme patterns are recorded from environmental influences or by mistakes through faulty DNA methyl editing machinery. For example, long-term type I diabetes (TID) complications are associated with a prior period of poor glycemic control, both micro- and macrovascular complications, including diabetic kidney disease, retinopathy, atherosclerosis, and vascular disease. These complications are predicted by a DNAme “metabolic memory”, detectable by specific CpG-DNAme changes decades before complications clinically present [6,7]. Several CpGs in combination can explain 68–97% of HbA1C association with the risk of complications development, with sites enriched genome wide for enhancers in blood and hematopoietic stem and progenitor cells (HSPCs), as well as open chromatin regions in myeloid progenitors [6]. Metabolic memory is predictive of elevated inflammation and vascular disease and may largely exist because stable DNAme changes encode a functional epigenetic history of extrinsic hyperglycemia in HSPCs, which is maintained by DNMT1 activity, and which drives functional defects in differentiated immune cells as well [6]. Therefore, DNAme signatures provide unique insights into developmental and environmental influences recorded on DNA as an epigenetic memory, even over vast stretches of time [9,36,37,38]. Given the essential role DNAme plays in development, it is not surprising that many cancers also routinely hijack DNAme networks for growth and metastatic behavior. CpG Island (CGI) hyperMethylation Phenotypes (CIMPs) and the reactivation of developmental gene networks are hallmarks of numerous cancers, frequently involving mutation or inactivation of DNMT or TET enzymes [29,39,40,41,42]. Other examples include promoter-CGI hypermethylation of the tumor suppressor MutL Homolog 1 (*MLH1*) in colorectal, esophageal, and thymic epithelial tumors [43,44,45,46,47] and promoter *TP53* DNAme in several cancers and in stroke patients [48,49,50]. Specific DNAme changes are also observed in atherosclerosis and cardiovascular disease (e.g., *ABCG1*), metabolic disorders (e.g., *ANKRD26*, *IGF2*), Alzheimer’s disease, and other nervous system disorders (e.g., *IGF2*) [36,37,51,52,53,54,55,56,57,58,59,60]. DNAme is highly predictive of biological age, and several pathological states accelerate DNAme age [6,9,10,37,38,61,62,63,64].

## 3. The Erasure of DNA Methylation from the Germline

Given the lifetime accumulation of environmental and age-associated epigenetic changes, mechanisms must exist to remove pathological epigenetic information where it exists in the germline. The timing of the first wave of demethylation is unique to male and female germ cells, and the second occurs shortly after male and female genomes have integrated. Germ line erasure overview. The mammalian germ line undergoes unique migratory, replicative, and global DNAme changes, which rearrange and epigenetically reconfigure the genome for its eventual passage onto the next generation. In mice, PGCs emerge from the proximal epiblast, numbering approximately 40 by 7.25 days post coitum (dpc) [65]. Migration across the posterior primitive streak to embryonic endoderm follows, with eventual migration to the genital ridge and gonad at 10 dpc [65,66]. It is during the migration and colonization of the genital ridges when global erasure of DNAme occurs, from ~70% to 4% at 13.5 dpc PGCs [15,66,67]. The X chromosome is reactivated, and imprinting sites are also generally demethylated [15,66,67]. Demethylation occurs in two phases, the first by passive demethylation from 8.5 dpc to 9.5 dpc, and active demethylation by TET1/TET2 from 9.5 dpc to 13.5 dpc, with peak 5mC oxidation activity at 11.5 dpc [12]. In males, acquisition of sex-specific DNAme occurs at 13.5 dpc forward, but female PGC DNAme patterns emerge after birth [12,66]. Specialized recombination will follow, allowing the separation of the diploid genome to haploid states as germ cells mature through meiosis [68,69,70,71]. Pre-implantation stages of DNA demethylation overview: Prior to fertilization, the male genome is at its highest level of CpG DNAme (~90%), and the female genome is near 40% DNAme [72]. The pronucleus enters the oocyte and begins to decrease DNAme even prior to the first round of division. An active process, TET3 is predominantly involved with the rapid conversion of 5mC to 5hMC and other derivatives, which persist until they are depleted by cell division; thus, demethylation is both active and passive [12]. The maternal genome is protected from high TET3 activity by maternal factor STELLA, and demethylation proceeds in a passive fashion [73]. Despite the virtual loss of all 5mC by the 16-cell stage of development, imprinting regions often clustered together escape this activity [14,74]. This is reinforced by low levels of site-specific DNMT1 activity and requires ZFP-TRIM28 heterochromatin-inducing activity to reinforce the marks [14,74,75]. Transposable element control relies heavily on histone H3 lysine 9 trimethylation (H3K9me3), with TRIM28/SETB1 controlling this deposition, along with other Krüppel associated box (KRAB) domain-containing zinc fingers, which promote eventual stable silencing by DNAme through recruitment of DNMT activity [12,74,76]. Additional insights may be gleaned by examining the behavior of imprinting sites and transposable elements during reversible cultures of naïve and primed pluripotent embryonic stem cells (ESCs), with the naïve state being hypomethylated relative to primed, more developmentally forward ESCs [32,77]. Given the natural occurrence of rare imprinting regions that naturally escape global demethylation events and augment the acquisition of global DNAme in PGC and pre-implantation blastocyst stages of development, accumulating evidence suggests some DNAme patterns are transgenerationally heritable.

## 4. Transgenerational DNA Methylation

**Figure 4:**
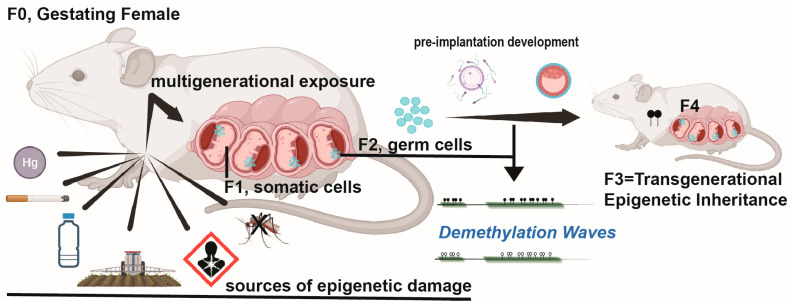
An overview of transgenerational inheritance. Potential sources of environmental exposure that may directly affect the gestating mother, developing F1 offspring, or migrating F2 germ cells within F1 offspring are displayed. The effects may also be indirect, such as F0 extreme trauma, which might alter hormone signaling and augment developmental signaling within the fetus and F2 PGCs. Phenotypic influence and epigenetic inheritance to the >F3 generation, without continued exposure to the original F0 event, is considered transgenerational.

It is now widely understood that genotoxic or epimutation events in embryonic development can influence lifelong propensities to develop wide-ranging human pathophysiological conditions. This is especially true during embryonic development spanning PGC migration and development, where both first-generation (F1) embryos and second-generation (F2) PGCs may be exposed to the same toxin or its effects. Thus, when considering a gestating mother, only the detection of the epimutation that drives a phenotypic change in the F3 generation, without continued exposure to the original insult, can be considered transgenerational (Figure 4). If, however, exposure of the postnatal mother or father (F0) results in F1 germline exposure, then the first unexposed transgenerational effect is F2 [78]. In either case, continued transmission of the epigenetic effect suggests transgenerational maintenance.

There is now considerable evidence for transgenerational epigenetic phenomena. Well demonstrated in plants, major environmental changes such as drought produce distinct epigenetic changes that can alter flowering rapidly and for generations [79]. For example, increased Lcyc promoter DNAme induces radial from bilateral symmetry, which is heritable for >100 generations [80]. Transgenerational epigenetic phenomena have since been observed in insects [81,82], zebrafish [83,84], birds [85,86], and mammals, including humans [87,88,89]. In mice, early demonstrations involved gestational exposure to the fungicide vinclozolin, which results in germline DNAme changes and sperm and fertility defects through F4 generations [90]. Since this landmark study, several additional environmental pollutants have been implicated in transgenerational disease. Major examples include pesticides containing DEET [91], mercury [83], tributyltin [92], insecticides [93,94], cigarettes [95], and plastic components including Bisphenol A (BPA) and phthalates, among others [94,96,97,98]. This also includes traumatic stress and famine [99,100]. These have been summarized in multiple reviews [94,101,102,103]. Effects can be wide-ranging and include reduced sperm counts and fecundity, kidney disease, obesity, immune dysregulation, cardiovascular disease, and cancer [45,97,102,104,105].

Malnourished mothers give rise to offspring with increased metabolic and cardiovascular disease incidence [106,107]. This so-called “thrifty phenotype” is increased obesity and poor adipose mobilization in offspring subjected to poor fetal nutrition [107]. This extends to adult F2 offspring of gestationally exposed F1 fathers, who had higher weights and body mass index (BMI) relative to unexposed F1 fathers from the 1944–1945 Dutch famine [88]. Several other studies have linked transgenerational effects from both overnutrition and malnutrition to metabolic syndromes and increased adiposity [108,109,110]. Interestingly, these effects are also observed with prenatal exposure to glyphosate [93], plastics [96], dioxins, and dichlorodiphenyltrichloroethane (DDT) [111]. Among dioxins, 2,3,7,8-tetrachlorodibenzo-p-dioxin (TCDD), the so-called “Agent Orange”, was widely utilized as a herbicide in the Vietnam War. It is considered the deadliest. At 0.1% lethal levels of oral F0 exposure, the unexposed F3 generation has significantly elevated levels of kidney disease. Depending on the study, dozens to hundreds of differentially methylated regions (DMRs) were induced and observed in the F3 generation [112]. Differential DNAme was noted at the progesterone receptor and insulin-like growth factor (Igf2), which may also extend to the imprinting control region [112,113,114]. Given the role Igf2 differential methylation may play in Alzheimer’s and major psychosis events, transgenerational DNAme at this locus may provide an epigenetic basis for potential neurological conditions associated with ancestral exposures [51,52,113]. For DDT, the transgenerational effects appear to be mediated through specific changes in sperm DNAme, with many DMRs that occur at genes known to promote obesity. F3 offspring were obese, with kidney disease in both males and females [111]. Interestingly, low-density regions termed ‘CpG deserts’, which contain <15 CpGs/100 bp but near clusters of CpGs, were identified to be associated with environmentally induced differential DNAme in sperm, which appears common to transgenerational DMRs [115,116]. These DMRs escape DNAme erasure during embryonic development, similar to imprinting sites [117].

## 5. Concerns for Global Health

Differential methylation effects on sperm DNA have been demonstrated to be toxin-specific [116]; therefore, unique environmental exposures, whether chemical, dietary, or otherwise, will likely manifest in specific human disease conditions or kinetics. However, the human reproductive period spans decades, lifespan decades longer, and in many instances, we are currently witnessing the health span effects on F3 individuals, observing the epigenetically heritable consequences of war, trauma, unregulated industrial expansion, and pollution in real-time. We are likely only beginning to understand the transmission of epigenetic information across generations, and certain profound effects may already be here. From 1973 to 2018, sperm counts have declined a staggering 62.3% from 101.2 million/mL to 49.0 million/mL, the rate only accelerating to 2.64%/year each year since 2000 [118,119]. It is unclear where the bottom of this decline is, but counts below 20 million/mL are considered low, with expected delays to conception. At trends of 2.64% declines/year (documented through 2018 [118]), this would occur in approximately the year 2053.

The link between reduced male fertility exposure to pollution, chemicals, and endocrine-disrupting agents is quite clear, and many of these established factors are now under transgenerational scrutiny [97,101,102,104,105,118]. However, it is likely a combination of one’s individual exposure, as well as intergenerational and transgenerational effects mediated through ancestral exposure, which are collectively driving a reduction in sperm counts. The extent to which inappropriate epigenomic changes are interactive, cumulative, and/or allowable for overall human reproductive viability remains to be determined. Murine studies suggest a single acute exposure to certain environmental toxins is sufficient. However, the ubiquitous presence of environmental toxins in our lives today, coupled with the biological magnification of environmental toxins within our food chain, feeding pathological information directly into the germline of exposed early embryos and PGCs, provides a basis by which humans are concentrating pathological epigenetic signatures within the genomes of unborn children, animals, insects, and plants, and potentially generations of offspring. This extends beyond fertility- to the potential inheritance of predisposing epigenetic events to cancer, diabetes, cardiovascular disease, diseases of the central nervous system, and the overall reduction of human life expectancy.

Thus, strategies must continue to be globally implemented and expanded to limit human exposure to genotoxic and epigenotoxic chemicals and pollutants, and events such as war, trauma, disease, and famine. Otherwise, the effects may persist for countless years, well beyond the generation of initial exposure, and we may increasingly rely on high throughput screening approaches and in vitro fertilization to alleviate the inheritance of epigenetic events that reduce human health and lifespan. In cases where pathological epigenetic information exists, retention may exist for several generations, potentially hundreds of years when considering human life expectancy; thus, epigenetic editing strategies that remove sites of epigenetic dismay may be increasingly needed.

## 6. Locus-Specific DNA Methylation Editing

**Figure 5:**
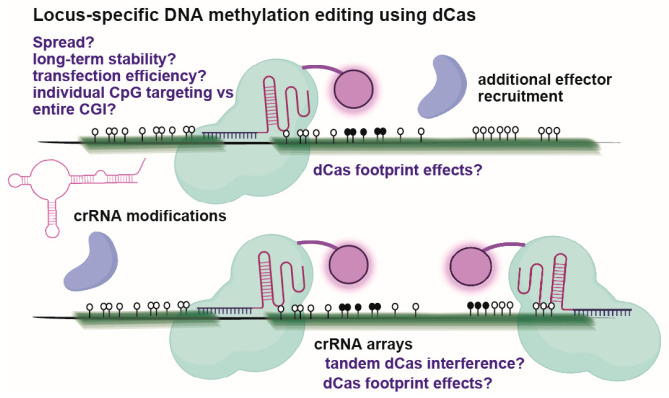
Overview of dCas-based DNAme editing. Briefly, enzymatically “dead” Cas enzymes are positioned adjacent to target CGs or CGIs for effector domain recruitment. Primary concerns for a typical experiment are highlighted in blue text, with additional potential modifications that may enhance editing capacity. Transfection and genome homing efficiency are critical factors for successful editing, as are effector domain selection, the timing of dCas, orthogonal system deployment, and the avoidance of immune responses for repetitive editing strategies.

Researchers have, for some time, fused epigenetic effector domains to homing proteins [120,121,122]. The CRISPR/Cas revolution facilitated the genome-wide examination of such approaches, in which nuclease deficient or enzymatically “dead”, CRISPR-associated protein 9 (dCas9) guided to DNA by CRISPR RNAs (crRNAs) can be fused to select epigenetic modifiers for locus-specific epigenetic editing (Figure 5). Typical Cas9 inactivation involves point mutations at each of the nuclease domains (D10A and H840A), and several orthogonal dCas versions now exist with unique sizes and guide RNA-DNA binding rules [123,124,125,126]. Functional effector groups include the catalytic domains of DNMT3a, histone acetyltransferase p300, and histone demethylase LSD1, among others [127,128,129]. dCas9-DNMT3a methylates CpG’s in a 25–35 bp peak but inhibits DNAme directly beneath the dCas9 footprint, and crRNA targeting is variable across entire CGIs [127,130,131,132]. Larger effector domain fusions or presentation strategies, such as KRAB, may enhance epigenetic editing activity and facilitate larger DNAme edits that are more compatible with CGIs [133,134,135]. For example, “CRISPR-off” uses a dCas enzyme fused to Znf10-Krab, Dnmt3A, and Dnmt3L protein domains, and depending on configuration, it can effectively induce long-term target gene suppression through promoter targeting [133]. “CRISPR-on”, by comparison, utilizes transactivator domains fused to MS2 coat protein, including VP64, p65-AD, and Rta, and recruits these domains via two MS2 stem loop sequences embedded within the crRNA [133,136,137]. Yet, most DNAme editing studies to date have been conducted with simple-to-transfect immortalized cell lines (i.e., 293T cells), and delivery of ever larger dCas fusions or scaffolds within single cells alongside dozens of crRNAs simultaneously is generally not compatible with efficient in vivo viral delivery systems [138]. Smaller dCas orthologs, such as from Staphylococcus aureus, have been used in adeno-associated virus (AAV)-mediated targeting of murine liver or mRNA-based delivery in HSPCs [139,140]. dCas9-SunTag has been used in transgenic mice for gene activation in the liver and midbrain [141,142]. Other transgenic versions include Rosa26:LSL-dCas9-p300 for gene activation and Rosa26:LSL-dCas9-KRAB for gene repression, with effects examined in the liver and the brain [138]. Immune responses to bacterial Cas proteins remain a concern for the long-term, repeat genome, and epigenome editing strategies [143,144]. For example, in mice with pre-existing exposure and immunity to SaCas9, liver genome editing by AAV delivery could occur, but the effect was accompanied by cytotoxic T-cell responses, hepatocyte death, and complete elimination of gene-edited cells [144]. Despite progress in locus-specific gene activation and repression of these systems, there are few reports on using these systems for DNAme editing during early developmental windows.

## 7. DNAme Editing in Germ Cells and Early Embryos

During both iPSC formation and rapidly upon somatic cell nuclear transfer (SCNT), the epigenome adopts a plastic, hypomethylated state. However, there are sites resistant to pluripotent or totipotent reprogramming; residual epigenetic signatures can reflect the original somatic cell of origin and be resistant to proper X-chromosome inactivation [145,146,147,148]. Thus, some forms of global correction have been explored, such as histone deacetylase inactivation with Trichostatin A, which also decreased DNAme and increased cloning efficiency [148,149,150,151,152]. Direct locus-specific epigenetic manipulation in early development has only been recently described. In oocytes, dCas9-TET1 or, separately, dCas9-DNTM3a were introduced as mRNAs by microinjection and used to edit premeiotic oocytes prior to ovulation. This resulted in DNAme editing at the intracisternal A-particle (IAP) repetitive promoter, which regulates the Agouti gene [153]. Coat color changes were noted in accordance with *IAP* promoter DNAme levels. The *H19* ICR was also targeted in a bimaternal mouse model with DNAme editing designed to mimic paternal imprinting states, resulting in recovering developmental competency [153]. Similarly, DNAme editing by zygote microinjection with dCas9-fused to an engineered prokaryotic DNA methyltransferase MQ1, targeting the *H19/Igf2* paternally imprinted locus, was reported to increase DNAme modestly at 2/5 crRNA test sites in offspring, with inhibition of DNAme beneath the dCas9 footprint [154]. The lack of additional targets, functional weight gain with H19 DNAme increases, or heritability testing suggests a lack of transgenerational effects with this approach, or at this location [154,155]. Likely due to known global erasure events in DNAme and/or the difficulty in delivering efficient epigenetic editing systems to germ cells and zygotes, there are no other reports on germ cell or early embryonic designer DNAme in mammals using dCas9 based editing. Next-generation epigenetic editing systems may need to incorporate factors involved in DNAme deposition in germ cells and transitions from naïve to primed pluripotency during blastocyst stages of development. For example, P-element-inducing wimpy testis (PIWI)-related protein, MIWI2, is critical for retrotransposon silencing via PIWI-interacting RNAs. Accordingly, a zinc finger-MIWI2 fusion induced DNAme and suppression of type A LINE-1 gene and rescued otherwise inhibited spermatogenesis in MILI-null mice [156]. Dnmt3l (and Dnmt3C in mice) is most highly expressed in germ cells and ESCs, where it exerts effects on imprinting control regions and transposable elements via DNAme. Many studies have indicated that Dnmt3l enhances Dnmt3A and 3B activity, though this may be region or site-specific and can be antagonistic depending on Dnmt3l interactions with PRC2 at bivalent promoters [157,158]. Additional updates to early developmental epigenetic editing systems may include guide RNA modifications or the recruitment of stage-specific co-repressor, and activator complexes involved in imprinting or repetitive element maintenance.

## 8. Transgenerational DNA Methylation Editing in Mammals

**Figure 6:**
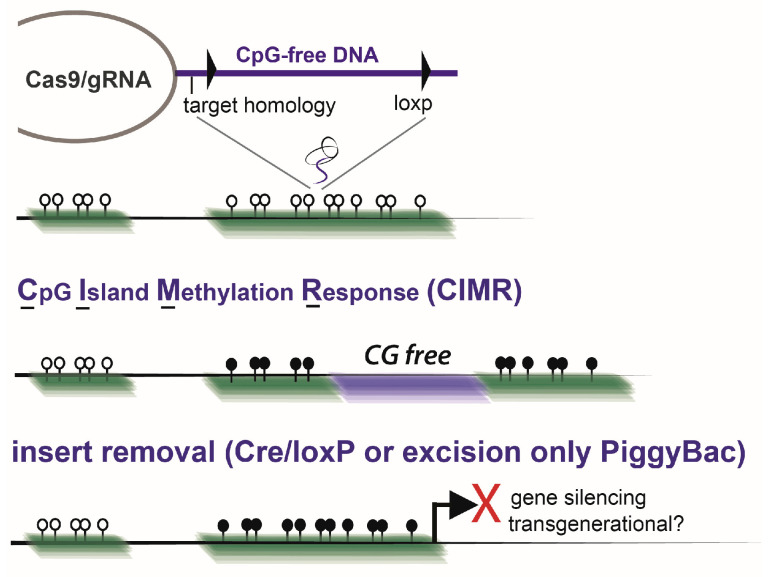
An overview of CpG Island Methylation Responses (CIMRs) that induce CGI-wide DNAme. Briefly, CG-free DNA is inserted into CG-dense CGI sequences (either double- or single-stranded DNA may be integrated). Uniquely, in primed pluripotency, the flanking CGI CpGs become spontaneously methylated. For most instances of promoter DNAme, this results in stable long-term gene silencing. DNAme is retained after CG-free insert removal and enables transgenerational testing of a single-engineered CGI by tracking DNAme in subsequent generations of offspring when tested in vivo.

Reminiscent of repetitive element silencing, Takahashi et al., recently identified that CG-free DNA insertion into CGIs triggered CGI-specific and CGI-wide de novo DNAme in both human PSCs and mouse PSCs [8,58] (Figure 6). Remarkably, mouse blastocyst/mPSC chimera offspring retained DNAme edits, which, despite the subsequent removal of the inserted DNA in mPSCs, was transgenerationally inherited [58]. Epigenetic obesity and hypercholesterolemia were mediated by targeted *Ankrd26* and *Ldlr* CGI promoter DNAme gains, of which designer DNAme patterns were not reduced until the F4 and F6 generations, respectively [58]. The re-occurrence of the methylation response, generation-to-generation, despite transient erasure during PGC development, suggests that induced transgenerational reacquisition of patterned DNAme is dependent on specific histone marks that re-adopt stable DNAme silencing upon transitions through later stages of embryonic development. H3K9me3 was observed to be increased in regions showing transgenerational behavior at target CGIs where DNAme was reacquired, despite transient erasure through germ cell development. Much like retrotransposon regulation depending on H3K9me3 deposition to induce de novo DNAme and stably silence elements, CGI Methylation Responses (CIMRs) may occur due to specific changes to CGI architecture that resemble viral insertion events. This may occur in response to the expected removal of CGI binding factors when the CGI is initially interrupted, which otherwise protects the CGI from DNAme editing machinery and premature differentiation-associated acquisition of DNAme. These observations may also be related to “bivalent domain” resolution during development, in which many developmental enhancers and promoters contain both activating and repressive marks, poised for rapid and coordinated expression response in differentiation [159,160]. When synthetic CGIs are introduced randomly into mESCs, they adopt a bivalent unmethylated state. Yet, when CGIs are integrated at high AT density, all CpGs become simultaneously DNAme, and bivalency is resolved. Furthermore, low-density deserts and CpG clusters, which appear to escape transgenerational DNAme erasure during responses to environmental toxins, appear to behave similarly to these effects [115,116]. Overall, programmable transgenerational DNAme editing is possible, but whether these approaches can be generalized genome-wide remains to be determined. We have similarly observed CG-free insertion by synthetic CpG-free ssDNA insertion in both human and mouse embryonic stem cells to induce CGI-wide, stable, and globally specific DNAme [8]. Functionally retained through in vitro differentiation, engineered DNAme is retained post-CG-free DNA removal and through multilineage differentiation. Furthermore, designer *MLH1* promoter DNAme was observed to skew thymic epithelial cell differentiation and sensitize multiple lineages to cisplatin [8]. By transcription factor binding enrichment analysis, we identified KLF6 as having a putative role in regulating CIMR responses. With regards to developmental CIMR timing, blastocysts did not exhibit targeted DNAme of the *Ldlr* CGI, but epiblasts did; thus, acquired DNAme at these interrupted CGIs occurs after implantation, upon re-establishment of global DNAme. In hESCs, we have observed CIMR DNAme acquisition to be restricted to the primed state of pluripotency and for reacquisition to occur when hESC transition from naïve to primed states using defined cultures ([8,15] unpublished observations). Collectively, this suggests that CG-free-DNA insertion into CGI or CG-dense areas of the genome, in germ cells or zygotes, will trigger locus- or region-specific DNAme as embryos transition through primed stages of development. In humans, this reflects pre-implantation blastocyst stages, and, in mice, this reflects the post-implantation epiblast stage. Regardless of the induction technique, the tracking of induced DNAme through inheritance, both with and without the continued presence of the original DNAme-inducing insert DNA, is essential for establishing transgenerational behavior. Whereas programmable transgenerational epigenetic transmission (PTET) involves designer epigenetic configurations that escape normal germline and pre-implantation stages of erasure and which influence the phenotype of subsequent generations, programmable transgenerational epigenetic reacquisition (PTER) involves epigenetic configurations that appear to be erased during PGC development and/or pre-implantation stages of development, but which are faithfully retriggered in each new developing embryo without the continued influence of epigenetic editing systems.

Mechanistically, transgenerational maintenance of programmed DNAme can be envisioned through the protection of acquired DNAme via germ line demethylation waves, in much the way STELLA, PGC7, or ZFP57 bind and protect imprinted genes. Alternatively, this may occur through the reacquisition of acquired transgenerational DNAme patterns with each ensuing generation, triggered iteratively through primed stages of pluripotency in each generation, in much the way CIMRs or specific forms of repetitive elements behave [8,58,161,162]. Demethylation strategies may be global, such as 5-aza-2′deoxycytidine and TSA, which non-specifically remove DNAme and have been used to improve SCNT cloning outcomes [151,152]; however, pathological sites of transgenerational DNAme could prove rare and highly specific, and thus warrant refinement of locus-specific demethylating strategies described above (see Locus-specific DNA methylation editing). Specifically, the use of a developmentally restricted effector or epigenetic editing domain presentation which naturally operates in germ cells or early embryonic development may be particularly helpful for removing stable sites of transgenerational memory.

**Figure 7:**
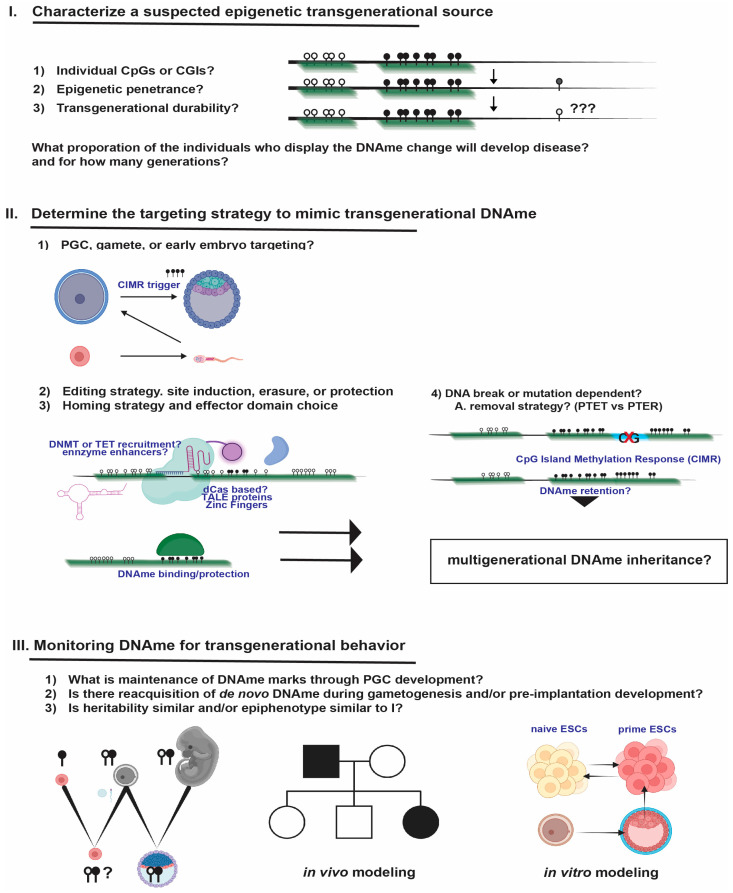
A basic framework for determining whether programmed DNAme is transgenerational has been provided. Major considerations for editing are provided as follows. (**I**) Characterize the source of transgenerational detriment, epigenetic penetrance, and the expected generational durability. Which DNAme sites are changed relative to normal, age, and sex-matched controls without exposure? What proportion of individuals with a particular heritable DNAme exhibit the negative epiphenotype? How many generations of transgenerational inheritance may be expected? For strongly modeled candidates or those supported by long-term epidemiological studies on transgenerational inheritance, epigenetic editing for mimicry can be used to test the suspected site for programmed transgenerational effects. Closed circles represent methylated candidate CG sites, which can be found in clusters or individually. (**II**) Determine the targeting strategy to mimic transgenerational DNAme. Targeting of PGCs, gametes, or early embryonic windows is preferred for inheritance into entire organisms, and for targeting developmental windows, which precede next-generation PGC migration and development for inheritance testing. Editing strategies are generally protein-homing-based and may also include certain adaptations to the enzymatically dead Cas systems, such as crRNA modifications and effector domain scaffolds. Specific domains or small RNA co-factors active during key developmental windows may enhance these activities towards transgenerational outcomes, especially those building histone marks, such as H3K9me3, which promote eventual DNAme deposition during later stages of development. Demethylation strategies generally rely on the recruitment of active demethylating enzymes or activating domains, such as VP64, to override DNAme. Engineered DNAme sites may also be protected by binding certain factors, such as STELLA, to prevent first-generation removal and allow for durability testing in subsequent generations. Insertions of CpG-free DNA or mutations that drive changes in transcription, for example, may induce local CGI DNAme spreading epimutations. Retained after the original insult, these naturally aid in transgenerational testing. (**III**) Monitor DNAme for transgenerational inheritance. Programmable Transgenerational Epigenetic Reacquisition (PTER) is the intentional, locus-, or region-specific induction of specific epigenetic configurations, which fail to escape germline or pre-implantation erasure but that are retriggered with each ensuing generation without the continued presence of epigenetic editing systems. This is unique from Programmable Transgenerational Epigenetic Transmission (PTET), which involves protection from germline and pre-implantation stages of epigenetic erasure. Either form of inheritance is of interest to human biology, but these remain important mechanistic clarifications, which can be further defined by stage-specific isolation of developing PGCs and gametes, developing blastocysts, and the examination of multigenerational inheritance in somatic tissues of offspring. Pedigree analyses can aid in establishing transgenerational transmission, penetrance, and durability. For in vitro modeling, which may include human pluripotent stem cells, the reversible cycling of cells between primed and naïve states enables reversible global DNAme switching and the testing of DNAme between additional generations of development. Sites or regions that wane in DNAme over repeat cycling, especially with DNAme-inducing DNA or mutations removed, are less likely to maintain transgenerational activity in vivo. For human early embryo studies modeled in vitro, the 14-day rule would be sufficient for understanding DNAme inheritance through primed stages of pluripotency and up to gastrulation. Beyond this, in vitro mimics of specific human somatic lineages remain state-of-the-art. Given the difference between humans and mice in repetitive element and imprinting regulation, human studies are preferred for human biology; however, preclinical modeling remains essential to understanding and testing transgenerational DNAme correction.

In closing, inducible early embryo DNAme editing, whether by “dead” homing protein-based or programmed de novo DNAme induction events, enables inheritance testing of tailored DNAme into all derivatives of the three germ layers and through the specification of germ cells. To facilitate continued discovery and corrective strategies for these DNAme conditions, readers are provided with a framework for inducing and testing specific sites of transgenerational DNAme inheritance (Figure 7).

## 9. Future Concepts and Ethics

Transgenerational DNAme induction is relevant to numerous human diseases with many conditions affecting F3 generation offspring living today. Several human disease states are predicted by relatively few specific DNAme changes, and thus, it would seem even rarer that similar DNAme sites would escape normal germline erasure. However, evidence points to these exact events. The inheritance of novel sites of intrinsic epigenetic regulation may simultaneously drive germ cell selection and, when maintained into embryonic development, skew certain cell behaviors in much the way an altered epigenetic landscape changes differentiation potential (Figure 2). There is some natural variability in sperm, egg, and early embryo DNA methylation profiles; these are important drivers for stochastic events in germ cell selection and embryo specification, respectively [153,163,164,165]. However, some sites of differential DNAme are clearly disruptive to fertility. This includes H19 imprinting centers and several male age-associated CpGs and DMRs [166,167,168]. DNAme epigenetic-age testing may be additionally informative when assessing reproductive fitness and applied to in vitro fertilization screening protocols. It is possible that certain levels of epigenomic rejuvenation through the expression of naïve pluripotency, germ-cell-specific transcription factors, or small molecule mimics may enable a reduction in sperm or egg biological age and improve rates of conception.

The rapid decline in male sperm counts is troubling. Evidence indicates environmental pollution, trauma, and malnutrition as sources of epigenetic change, leading to declining germ cell function. Thus, germline or early embryo epigenetic editing may increasingly be warranted to remove disease-predisposing transgenerational information. Strategies are outlined in Figure 7. For some cases, single CGI or CG edits may be needed, requiring single-cell, single-DNAme-molecule read technologies for high-confidence selection of edited cells [153]. In the future, we may consider site-specific editing at dozens to thousands of sites simultaneously for activation, silencing, poising, and enhancing, all in coordination with the timing of extrinsic developmental signals. This form of systems-level deterministic epigenome manipulation will require significant development to safely alleviate transgenerational disease. The human lifetime is long, and perhaps even capable of extension through epigenomic rejuvenation, but accurately assessing lifespan extension can be challenging [169,170]. Epigenetic clocks that accurately predict biological age, aging rates, and disease propensity will, thus, play an important role in understanding whether certain toxins or events instill transgenerational DNAme, driving aging and multigenerational disease [61,63,171]. One may envision similar clocks applied to assessing transgenerational age or disease acceleration through the examination of specific sites or combinations of sites that persist across generations.

This review presents an overview of the literature that has implicated transgenerational DNAme in the reacquisition of certain pathophysiological conditions across generations. Strategies have also been presented for editing germline and early embryo transgenerational DNAme. Indeed, a world where the residual epigenetic effects of trauma, famine, and pollution have been erased from our germ line may very well be more peaceful and filled with longer life. This, of course, depends on the extent to which epigenetic changes can be deterministic. However, despite our technological leaps, mammalian and human development, especially post-implantation stages of development, still harbors an incredible mystery. For example, transgenerational DNAme may ostensibly arise from co-segregation with a mutation in a neighboring gene, which removes transcription termination [172,173]. When transcription was mutationally extended, de novo DNAme was also extended as it does across actively transcribed regions, and this induced promoter DNAme of the adjacent gene [172]. Thus, primary or secondary effects of mutation or DNAme are not always initially clear; even pinpointed DNAme edits early in life may have unexpected consequences among the trillions of derivative cells much further in life. Therefore, much like gene editing, developmental human epi-gene editing beyond accepted in vitro culture limits should also be under similar moratorium considerations [174].

## 10. Summary

Transgenerational epigenetic influences exist across plant and animal species, drive evolution, and may accelerate adaptive responses to detrimental environmental events. However, these processes occur during developmental windows, which are highly sensitive to environmentally, nutritionally, or hormonally induced changes to the genome and epigenome, of which gestational windows may simultaneously affect both F1 and F2 generations. Here, certain epigenetic insults or epigenetic edits may be transgenerationally durable. Recent advances in early embryo editing have enabled transgenerational disease modeling mammals for the first time. In a future where evidence for human transgenerational disease mounts, technologies may be needed that stably correct generations of otherwise reoccurring epigenetic disease.

## Abbreviations

5mC	5-methylcytosine
CpG	cytosine-phosphate-guanine
DNAme	DNA methylation
PGC	primordial germ cell
ESC	embryonic stem cell
DMR	differentially methylated region
DNA methylation	The covalent addition of methyl groups to DNA. Though this predominately occurs at 5′-CpG dinucleotides, non-CG methylation is noteworthy in pluripotency and various neural lineages
Epigenetics	The cellular and organismal heritability of internal factors, including the modifications to them and by them, those recorded from environmental influences and in developmental history, whether physically local to the cell, signaled across an organism, or accumulated from sources larger in nature (for example, hormones, pollution, viruses, diet, and lifestyle), that influence the expression of chromosomally associated genetic information, establish stable cellular states over differentiation (or unstable states in aging and pathology), and enable the physical, biochemical, behavioral, cognitive, and social nature of an organism to emerge and function, without altering the primary DNA sequence [20]
Epimutation	An alteration to an epigenetic factor, such as DNA methylation, which may occur in response to an environmental insult or via incorrect deposition of the epigenetic feature
Programmable Transgenerational Epigenetic Reacquisition (PTER)	The intentional, locus- or region-specific induction of specific epigenetic configurations that fail to escape germline or pre-implantation erasure, but which are retriggered with each ensuing generation without the continued presence of epigenetic editing systems
Programmable Transgenerational Epigenetic Transmission (PTET)	The intentional, locus- or region-specific induction of specific epigenetic configurations that escape germline erasure and influence the phenotype of subsequent generations without the continued influence of epigenetic editing systems
Transgenerational Epigenetic Inheritance	The transmission of epigenetic information across generations that escapes germline erasure
